# World Heavyweight Championship boxing: The past 30+ years of the male division

**DOI:** 10.1371/journal.pone.0263038

**Published:** 2022-01-24

**Authors:** Mitchell James Finlay

**Affiliations:** Sports Injury Research Group, Department of Sport & Physical Activity, Edge Hill University, Ormskirk, Lancashire, United Kingdom; Nottingham Trent University, UNITED KINGDOM

## Abstract

Data from the past 30+ years of the male boxing World Heavyweight Championship (n = 182 bouts) was obtained. The USA were the most represented and produced more champions than any other nation, followed by the UK, Ukraine and Russia. Denmark (100%), Ukraine (85.4%) and the UK (67.3%) produced the greatest ‘success rates’ in World Heavyweight Championship contests. Where possible, comparisons between bout winners and losers were also made. Winners were significantly taller (*p* < 0.001, *d* = 0.35) and had a greater reach (*p* = 0.003, *d* = 0.23) when compared to losers. Championship bouts were settled by the following methods: a form of knockout (101), points decision (57), retirement (14), draw (3), disqualification (3), technical decision (1), whilst 3 no contests were omitted from the analysis. Total punches thrown and landed, and jabs and power punches thrown and landed were consistently significantly greater (*p* < 0.005, *d* = 0.27–0.73) in winners, compared to losers. Winners were more accurate compared to their losing counterparts by ~ 8 percentage points. The data presented in the present study clearly show some anthropometric advantages of championship bout winners, compared to their unsuccessful counterparts, and that winners are more active and accurate when compared to losers. The punch output data, albeit very basic, may be useful in informing tactical strategy and preparation of heavyweight prospects. Likewise, the data in the present study may be an interesting resource for professional boxing enthusiasts. Future research should seek to replicate the analysis in the present study across other weight divisions to explore any potential differences between weight classes. Additionally, extending the analysis to female boxing may provide interesting comparisons.

## Introduction

As one of the 8 original professional divisions, the heavyweight boxing division has long been established as one of the most popular divisions in boxing. A unique element of heavyweight boxing is that there is no upper limit on body mass, resulting in less constraints when compared to boxers in other weight divisions [1, 2]. The greater stature (compared to other divisions), and the greater focus on winning by knockout (KO) may be contributing factors to the division’s popularity, although the latter may be anecdotal with no apparent performance analysis data comparing KO’s in professional boxing by division. Nonetheless, modern day heavyweight champions enjoy global recognition that can transcend the sport, thus heavyweight boxing has the potential to amass large revenues [3]. Despite the popularity of the heavyweight division, there is little descriptive information in the scientific literature on contests for an internationally recognized version of the World Heavyweight Championship. This is limited to only one recent study exploring the anthropometric trends of heavyweight boxers since the inception of a World Heavyweight Championship [1]. It was reported that alike other sporting populations [4, 5], heavyweight boxers have increased in size as determined by body mass index (BMI) and reach, over the past 130 years [1]. The authors reported that champions were taller (3.4 cm; *p* < 0.001), heavier (+3.7kg; *p* = 0.017), and had a longer reach (+3.6 cm; *p* = 0.005) compared to their challenger counterparts. The authors also reported the mean age of championship contestants was 28.9 years, which did not differ significantly between winners and losers. Even less is known about boxer background, their stance, or their record at the time of a championship bout. Only one study, the same as above, reported the nationality of World Heavyweight Championship contestants [1], revealing that the USA was the nation with the most World Heavyweight Champions.

Several performance analysis studies in amateur boxing [6–12] have allowed for the quantification of the external demands of the sport, and assisted in the development of boxing-specific simulations based on notational data [13–17]. Specifically, in a boxing context, the number of attacking and defensive maneuvers, as well as many other variables, can be quantified via slow-motion video analysis. This can be useful for boxers and their coaches in devising tactical strategies and improving future performances. Such analysis has shown that elite amateur boxers typically throw between 55–78 punches per round [7–9, 12]. However, in comparison to the amateur format, only a select few articles have been conducted in professional boxing [18, 19], with a lack of data on punch activity available.

In contrast to the 3 x 3-minute format of amateur boxing [12], professional boxing, in particular world championship boxing, lasts up to 12 x 3-minute rounds [2]. This would certainly require a greater total punch output. Although no comprehensive notational data on professional heavyweight boxing exists in the literature, Compubox [20], a well-known and long-standing provider of live statistics on professional boxing, have provided a more basic analysis of punch output since 1985 [21]. Specifically, two ringside operators count jabs and power punches thrown and landed by round and cumulatively over a bout [21, 22]. There are many limitations associated with the Compubox analysis, chiefly this relates to its accuracy and reliability in classifying punch output [21]. The classification of punches may also be elementary in comparison to the above studies in amateur boxing. Compubox separates punch output into only 2 categories, jabs and power punches, and does not include any of the additional information on defensive activity that the amateur boxing literature encompasses [7–9, 12]. Further, Langholz [21] notes that this live analysis, as opposed to analysing video retrospectively, may be susceptible to human error and bias.

Irrespective of the limitations, Compubox is the single method of professional boxing analysis that spans more than 30+ years, and therefore could provide novel and interesting information on the past 30+ year history of the World Heavyweight Championship. Data on the background, characteristics and anthropometry of heavyweight title contestants, the number of KO/points victories, and the types of punches thrown and landed, could provide interesting data for researchers and practitioners working with professional heavyweight boxers. Identifying the above potential differences between winners and losers could even be used for talent identification, training, or bout tactical strategies. This study could also offer initial descriptive information on punch output, as part of further comparisons between weight divisions, or between male and female boxing. This may also be of interest to professional boxing enthusiasts. Therefore, this study aimed to collate descriptive information on World Heavyweight Championship bouts and contestants of the past 30+ years, in addition to comparing data between winners and losers where possible.

## Methods

### Procedures

Bout outcome and data relating to the background and characteristics of boxers at the time of a World Heavyweight Championship bout, were obtained and cross-checked from the following publicly available internet sources; BoxRec [23] and Wikipedia [24]. Likewise, punch activity was retrieved and cross-checked from the following publicly available internet sources; BoxRec [23], Boxing Scene [25], and CompuBox [20]. Such approaches have been utilised in the analysis of mixed martial arts (MMA) bouts [26]. All data was concerned with official contests for an internationally recognised World Heavyweight Championship title (WBA, WBA Regular, WBO, WBC, IBF) in the male format, from April 1991 to January 2022. Though not one of the four major professional boxing organisations, data from IBO title bouts was also considered, as many marquee names in the heavyweight division have, and continue to hold the title to this day. Information was gathered for all competitors and further comparisons between winners and losers were made. Specifically, data from 182 bouts were initially collated. Where bouts were ruled as a no contest (3), data was omitted from the overall analysis. Therefore, 179 bouts were included in the overall analysis of bout outcome and boxer background and characteristics. A further 3 bouts were scored as a draw and were therefore omitted from the winners vs losers analysis, resulting in a total of 176 bouts included in the comparative analysis. The above analyses of championship bouts comprised 123 individual boxers. In instances where anthropometric data was unavailable for 1 or both boxers involved in a championship bout, data from such bouts were omitted from the analysis. Punch output data was not available for 41 bouts, therefore, a total of 135 bouts were included in the punch output analysis. The study did not use human subjects and all data was publicly available, therefore, ethical approval was not required for this research.

Where possible, the following information was gathered for all boxers, and for winners vs losers comparisons of championship bouts.

#### Boxer background and characteristics

Information on boxers nationality, age, anthropometry (height, reach and body mass), boxing stance, and boxing record at the time of a championship bout was collated.

#### Bout outcome and punch output

Bout outcome and punch output data in championship bouts, including; punches thrown, punches landed and punch type, was collated. The percentage of punches landed compared to punches thrown, and the percentage of jabs to power punches (defined by Compubox as any punch other than a jab) thrown were also calculated.

### Statistical analyses

All data was initially inputted to a large Microsoft Excel data sheet to obtain mean ± standard deviation (SD) and percentage values for all boxers, and for winners and losers. A Shapiro-Wilk test was conducted to determine the normality of data, which verified that the data was non-normal. Therefore, a non-parametric test (Mann-Whitney U) was used to compare differences between winners and losers of World Heavyweight Championship bouts. Initially, the distribution of differences was assessed for symmetry, and subsequently median values were obtained. A chi-square test for association was also performed to determine potential associations with bout outcome and boxing stance. Cohen’s d (*d*) effect sizes for the Mann-Whitney U tests were calculated by dividing the absolute z statistic by the square root of N, with the following thresholds: small = ≥ 0.2; moderate = ≥ 0.5; and large = ≥ 0.8 [27]. All statistical analyses were conducted using IBM SPSS Statistics for Mac, version 25 (IBM Corp., Armonk, N.Y., USA), with statistical significance set at *p* < 0.05.

## Results

### Boxer background and characteristics

Table 1 highlights the number of times a nation was represented, victorious and unsuccessful in a World Heavyweight Championship bout, in addition to the number of individual champions. USA was the most represented nation (164, 46.3%) in championship bouts, followed by the UK (55, 15.5%), Ukraine (48, 13.6%) and Russia (23, 6.5%). The number of winners in World Heavyweight Championship bouts followed a similar order. The majority of individual champions were of USA nationality (26, 52%), followed by the UK (7, 14%), Russia (5, 10%), Ukraine (3, 6%) and New Zealand (2, 4%). Nations with the highest success rates (percentage of wins from championship bouts) were Denmark (100%) and Ukraine (85.4%), followed by the UK, (67.3%), Russia (65.2%), and New Zealand and Uzbekistan (62.5%).

**Table 1:** Nationality of all contestants, winners, and losers of World Heavyweight Championship bouts.

Nation	Involved in a championship bout		Winner in a championship bout		Loser in a championship bout		Draw in a championship bout		Success rate	Individual champions	
USA	164	46.3%	62	35.2%	98	55.7%	4	67%	37.8%	26	52%
UK	55	15.5%	37	21%	16	9.1%	2	33%	67.3%	7	14%
Ukraine	48	13.6%	41	23.3%	7	4%	0	0%	85.4%	3	6%
Russia	23	6.5%	15	8.5%	8	4.5%	0	0%	65.2%	5	10%
New Zealand	8	2.3%	5	2.8%	3	1.7%	0	0%	62.5%	2	4%
Uzbekistan	8	2.3%	5	2.8%	3	1.7%	0	0%	62.5%	1	2%
Poland	8	2.3%	0	0%	8	4.5%	0	0%	0.0%	0	0%
Germany	7	2%	0	0%	7	4%	0	0%	0.0%	0	0%
Denmark	6	1.7%	6	3.4%	0	0.0%	0	0%	100.0%	1	2%
South Africa	5	1.4%	1	0.6%	4	2.3%	0	0%	20.0%	1	2%
Cuba	5	1.4%	0	0%	5	2.8%	0	0%	0.0%	0	0%
Mexico	3	0.8%	1	0.6%	2	1.1%	0	0%	33.3%	1	2%
France	3	0.8%	0	0%	3	1.7%	0	0%	0.0%	0	0%
Belarus	2	0.6%	1	0.6%	1	0.6%	0	0%	50.0%	1	2%
Bulgaria	2	0.6%	0	0%	2	1.1%	0	0%	0.0%	0	0%
Australia	2	0.6%	1	0.6%	1	0.6%	0	0%	50.0%	1	2%
Syria	2	0.6%	1	0.6%	1	0.6%	0	0%	50.0%	1	2%
Canada	1	0.3%	0	0%	1	0.6%	0	0%	0.0%	0	0%
Romania	1	0.3%	0	0%	1	0.6%	0	0%	0.0%	0	0%
Haiti	1	0.3%	0	0%	1	0.6%	0	0%	0.0%	0	0%
Jamaica	1	0.3%	0	0%	1	0.6%	0	0%	0.0%	0	0%
Costa Rica	1	0.3%	0	0%	1	0.6%	0	0%	0.0%	0	0%
Uganda	1	0.3%	0	0%	1	0.6%	0	0%	0.0%	0	0%
Italy	1	0.3%	0	0%	1	0.6%	0	0%	0.0%	0	0%

Table 2 presents descriptive data on the characteristics, anthropometry, and bout record at the time of a World Heavyweight Championship contest of all competitiors, and for winners and losers.

**Table 2:** Characteristics, anthropometry and prior bout data of all contestants, winners, and losers in World Heavyweight Championship bouts.

	All	Winners	Losers	Difference
Characteristics and anthropometry				
Age (years)	31.8 ± 4.7	31.2 ± 4.3	32.3 ± 5.1	1.1
Height (cm)	192.6 ± 6.5	194.3 ± 6.8	190.9 ± 5.9	3.4
Reach (cm)	200.8 ± 8.4	202.1 ± 8.3	199.1 ± 8.1	3
Body mass (kg)	107.9 ± 8.7	108.5 ± 9.3	107.4 ± 8.1	1.1
Record at time of bout				
Bouts	35.2 ± 11.9	35.4 ± 12.1	35 ± 12	0.4
Win %	94.2%	96%	92.6%	3.4%

Data are mean ± SD and % where applicable.

Mann-Whitney U tests revealed that winners were signficantly taller (*U* = 11006, z = -4.589, *p* < 0.001, *d* = 0.35) and had a greater reach (*U* = 11906, z = -2.970, *p* = 0.003. *d* = 0.23) when compared to losers. Median height and reach values of 196 cm and 201 cm, and 190.5 cm and 198 cm were observed for winners and losers, respectively.

There were no signficant differences betweeen winners and losers in age or body mass. Both winners and losers had a similar number of bouts at the time of a championship bout, whilst winners had a slightly higher percentage (+3.4%) of wins.

There were 321 instances where a World Heavyweight Championship bout comprised an orthodox boxer, compared to just 37 instances of a southpaw competitor. This translated to an 89.7% orthodox and a 10.3% southpaw boxer representation. A chi-square test for association revealed there was no statistically significant association between success in a World Heavyweight Championship bout, and boxing stance.

### Bout outcome and punch output

A knockout in the form of either a Technical knockout (TKO) or knockout (KO), was the most frequent bout outcome (101), followed by points decision (57), retirement (14), draw (3), disqualification (3) and technical decision (1). No contests accounted for 3 bouts and were omitted from the analysis.

Table 3 presents descriptive data on the punch output of all competitors, and by winners and losers in World Heavyweight Championship bouts. Winners landed a greater percentage of punches thrown compared to losers, by ~8 percentage points. Likewise, winners landed a greater percentage of jabs and power punches thrown, when compared to losers, by ~7 and ~10 percentage points, respectively.

**Table 3:** Punch output of all contestants, winners and losers in World Heavyweight Championship bouts.

Bout activity	All	Winners	Losers	Difference
Total punches thrown	320.1 ± 194.9	363.7 ± 209.1	271.3 ± 170	92.4
Total punches landed	110.9 ± 83.2	138.7 ± 88.7	81.1 ± 64.9	57.6
% Punches landed	34.6%	38.1%	29.9%	8.2%
Ave thrown per round	37.6 ± 15	44 ± 15.1	31.1 ± 12.3	12.9
Ave landed per round	13.3 ± 8.3	17.3 ± 8.7	9.3 ± 5.8	8
Total Jabs thrown	172.4 ± 123.7	197.2 ± 135.6	145.3 ± 107	51.9
Total Jabs landed	48.1 ± 43.1	60.8 ± 46.6	34.5 ± 33.6	26.3
% Jabs landed	27.9%	30.8%	23.7%	7.1%
Total Power thrown	147.8 ± 97.4	166.3 ± 102.5	126.4 ± 88.2	39.9
Total Power landed	62.7 ± 50.8	77.9 ± 55	46.6 ± 40.7	31.3
% Power landed	42.4%	46.8%	36.9%	9.9%
Jab/Power thrown %	53.8/46.2	54.2/45.8	53.5/46.5	-

Data are mean ± SD and % where applicable.

Mann-Whitney U tests revealed that winners threw and landed significantly more total punches (*U* = 6759, z = -3.669, *p* < 0.001, *d* = 0.32; *U* = 5499.5, z = -5.632, *p* < 0.001, *d* = 0.48) than losers, with median values of 371 and 134, and 276 and 64 for winners and losers, respectively. Likewise, average punches thrown and landed per round were significantly greater in winners (*U* = 4401, z = -7.344, *p* < 0.001, *d* = 0.63; *U* = 3668, z = -8.487, *p* < 0.001, *d* = 0.73) when compared to losers, with median values of 42.5 and 15.2, and 29.8 and 7.6 for winners and losers, respectively.

Mann-Whitney U tests revealed that winners threw and landed significantly more jabs (*U* = 7030.5, z = -3.245, *p* < 0.001, *d* = 0.28; *U* = 5777.5, z = -5.199, *p* < 0.001, *d* = 0.45) than losers, with median values of 194 and 53, and 134 and 24 for winners and losers, respectively.

Mann-Whitney U tests revealed that winners also threw and landed significantly more power punches (U = 7075.5, z = -3.175, *p* = 0.001, *d* = 0.27; U = 5804, z = -5.157, *p* < 0.001, *d* = 0.44) than losers, with median values of 155 and 67, and 113 and 36 for winners and losers, respectively.

## Discussion

The present study provided the first data on the bout outcome and punch output involved in World Heavyweight Championship bouts of the last 30+ years. This study also provided descriptive information on the background characteristics and anthropometry of championship contestants. Additionally, where possible the above information was used to identify differences between winners and losers. Championship bout winners were taller and had a greater reach when compared to losers; however, only marginal and non-significant differences in age or body mass were found. Likewise, the bout record at the time of a championship contest was similar between the two groups, though winners had a slightly higher percentage of wins. The USA were clearly the most represented nation in championship bouts and thus, produced the most individual champions. A TKO or KO was the most frequent method of victory in championship bouts, with points decision also a common bout outcome. The present study found that winners both threw and landed more punches than their unsuccessful counterparts.

### Characteristics and anthropometrics

The mean age of contestants for the World Heavyweight Championship was 31.8 ± 4.7 years, this ranged from 22 years to 47 years. The mean age and ~35 previous contests of contestants at the time of a championship bout reflect the considerable amount of time it takes for a heavyweight to reach the pinnacle of the sport. As no data from other divisions is currently available, it is not possible to compare the above findings with championship bouts across other weight divisions. A large majority (~90%) of boxers competed in an orthodox stance, though it is not uncommon for some boxers to switch between orthodox and southpaw if adequately skilled. The mean height and body mass of championship contestants was 192.6 ± 6.5 cm and 107.9 ± 8.7 kg respectively. The tallest contestant stood at 213 cm, whilst two boxers shared the shortest height of 178 cm. The heaviest recorded weigh-in was 148.8 kg, and the lightest 87.5 kg. The latter represents the rare event of an undisputed light-heavyweight world champion, moving up to challenge for the World Heavyweight Championship. There is clearly potential for large variations in the body mass of two opponents during a heavyweight bout, in a division with no upper limit on body mass [1]. Boxers in the present study were heavier than that reported previously [1], though this can be explained by the present study analysing the past 30+ years, whereas Han et al. reported the anthropometric trends of heavyweight championship contestants over a 130-year period. The authors reported that the body mass of boxers had increased over this time-period [1], in line with other athlete populations [4, 5]. Boxers reach in the present study was also greater than that reported by Han et al., though again, this is in agreement with the authors finding of considerable growth in heavyweight championship contestants over the past 130 years [1].

Previous literature suggest that the USA are the dominant nation of the World Heavyweight Championship since its inception [1]. It is therefore unsurprising that the present study found the USA were the most represented and produced the most individual champions (26) in the past 30+ years. The USA does not however, have one of the higher success rates in World Heavyweight Championship bouts. This may simply be a product of their much larger representation compared to other nations, where boxers from the USA are more likely to face each other. Other nations with considerable involvement in World Heavyweight Championship bouts are Ukraine, Russia, and the UK, though it is important to apply some context. Most of the above nations have had several championship competitors in the past 30+ years, whilst Ukraine’s inclusion is largely due to having two of the top 10 longest championship reigns in heavyweight history. Indeed, Ukraine has the second highest success rate of all nations, inferior only to Denmark, where a single competitor was victorious in all 6 of their IBO World Heavyweight Championship bouts. The UK and Russia both have a success rate of >60% in their championship bouts, suggesting they are also key nations in the history of the World Heavyweight Championship over the past 30+ years.

### Bout outcome and punch output

Findings of the present study show a majority of bouts were settled via a form of knockout, whether by TKO or KO. Heavyweight boxing has long been associated with an increased focus on KO’s and stoppages, though without across division comparisons, this remains anecdotal. Considering Newton’s second law of motion (F = ma), the increased body mass of heavyweight boxers may result in greater force production capabilities when compared to other divisions, as observed in amateur boxing [28, 29]. Loturco and colleagues [30] reported a large correlation (r = 0.58) between body mass and punch impact force in Brazilian elite level amateur boxers. However, the authors noted that heavier boxers may not necessarily produce more force in a punch, but that stronger and more powerful athletes may be more likely to achieve a KO. Indeed, in that particular study, strong correlations between strength and power capabilities and punch force (r = 0.67 to 0.85) were found. This highlights the importance of neuromuscular capabilities in boxing. Heavyweight boxers are in a unique position, whereby any training methods that may intentionally or inadvertently induce hypertrophy (such as different phases of strength and power training), are not to the detriment of ‘weight making’ requirements. Atha et al. reported the punch force of a rear cross from a single elite professional heavyweight boxer at 4096 N [31], which is greater than that reported in a professional super middleweight boxer (~3500 N) [32]. Data from several studies on amateur boxers [14, 29, 33] show an average rear cross punch force of ~ 3100 N, and ~ 2500 N for all punch types. However, comparison of punch forces between professional and amateur boxers, or between weight divisions, is difficult due to the different punch types analysed and the methods used to quantify such punches. Furthermore, the lack of research exploring the punch force of professional boxers with an appropriate sample size, means comparisons should be made with caution. Nevertheless, the fact that ~56% of bouts were settled via a form of knockout, may partly explain the lure of the heavyweight division.

The present study found World Heavyweight Championship contestants threw 37.6 ± 15 punches per round. Notational bout data on professional boxing is scarce; however, comparisons with the amateur format may again be useful. Elite amateur boxers typically throw between 55–78 punches per round [7–9, 12]. The differences may be partly explained by the inclusion of lower weight divisions in the amateur boxing literature. Likewise, differences in the punch output could be attributed to pacing strategies. Specifically, the professional format in championship bouts is 12 x 3-minute rounds, in contrast to a maximum of 3 x 3-minute rounds in amateur competition. Previous research has identified that pacing strategies may be employed in amateur boxing to maintain task-specific performance and potentially offset fatigue [9, 34]. Therefore, it could be speculated that pacing strategies may be more appropriate during the much longer duration of the professional format. Boxers in the present study landed 34.6% of all punches, and the percentage of jabs/power punches thrown showed a slightly greater tendency towards jabs. Elite amateur boxers land between 13–29% of the total punches thrown [8, 9, 12], which could suggest heavyweight professional boxers are more accurate than the elite amateur. The increased duration and the potentially increased importance of pacing strategies in professional heavyweight boxing, may allow for a more patient approach, with implications for punch accuracy. However, this could also represent differences in skill level between the two formats, with consideration to the elite level bouts analysed in the present study. Differences in the method of data collection, weight divisions, and skill levels, means comparisons between amateur boxing and the data in the present study should be interpreted with caution. Lastly, the lack of notational data or research on pacing in professional boxing, unfortunately means comparisons across weight divisions in professional boxing is not possible.

### Comparisons between winners and losers

The present study found several differences between winners and losers in World Heavyweight Championship bouts. The data suggest that taller boxers with longer upper limbs may have an advantage over shorter counterparts, in agreement with previous research [1]. In boxing, height and reach are desirable characteristics that may make a boxer more elusive or may be advantageous in managing distance via the jab. To use a phrase commonly used in boxing, many shorter boxers find it difficult to ‘get under’ the large reach of a taller boxer, thus minimising the effectiveness of their attacks. The marginal and non-significant age difference between winners and losers, was in agreement with previous work [1], but in contrast with other literature [35]. In contrast to the findings of Han et al. the present study found only marginal and non-significant differences in body mass between winners and losers [1]. As mentioned earlier, this may be due to the clear trend of growth in heavyweight boxers since its inception, with most heavyweights in modern boxing being much heavier than the 91 kg limit. At the time of writing, a new weight division termed ‘Bridgerweight’, is being considered by one of the leading sanctioning bodies [36]. This new division aims to ‘bridge’ the gap between the heavyweight division and the nearest division below it. It would be interesting to observe whether the adoption of this new weight class, could influence the winner vs loser differences in anthropometry found in the present study.

Data in the current study does not suggest that there is an advantage for southpaw boxers over orthodox boxers, in World Heavyweight Championship boxing. A commonly held view in boxing is that competing against a southpaw boxer may present a more difficult task than competing against an orthodox boxer. This view chiefly relates to there being fewer southpaw boxers [37], a theory confirmed by only ~10% southpaw representation in title bouts in the present study. This would inevitably mean there is less opportunity to train against boxers with an opposite foot and hand positioning [37]. Two studies in amateur boxing [10, 38] have concluded an advantage for southpaw boxers over orthodox boxers; however, both studies analysed small sample sizes, with one study analysing mixed ability boxers from a single gym [38]. Therefore, it is difficult to compare the findings of the current study to that of previous research. Nevertheless, boxing stance may not be a key factor in World Heavyweight Championship boxing in the past 30+ years.

The present study also found that the total and average punches thrown per round were greater in winners, when compared to losers. Likewise, the data in the current study show a greater accuracy in winning boxers at ~8 percentage points, when compared to losers. The percentage of jab/power punches thrown was similar between winners and losers, which does not support the adoption of a particular strategy (e.g., throwing more jabs or power punches) to increase the chance of winning.

### Limitations and future research

The punch output data in the present study was taken from publicly accessible websites, initially produced by a renowned punch analysis company. Punches are categorised into jab and power punches. As previously mentioned, this does not differentiate between hooks, uppercuts, or rear crosses. It also does not offer defensive data, in contrast to more advanced, retrospective methods of boxing notational analyses in the literature [7–9, 12]. Future research could provide a more detailed analysis of punch types, and other boxing performance analysis metrics involved in World Championship level professional boxing, such as defensive actions. This more in-depth performance analysis would further the understanding of the external demands of professional heavyweight boxing, and may in turn, influence training and bout strategies. This could also be extended further into other weight divisions or to female boxing, to enable across division or between-sex comparisons. Another limitation of the present study, although not in the control of the author, is that punch data was not freely available for 41 bouts. Lastly, there is no publicly available data on the reliability of the variables shown in Table 3, therefore the results should be interpreted with caution. Nevertheless, this represents the only available punch output data on professional boxing spanning across several decades and may serve as an interesting analysis for professional boxing practitioners or enthusiasts.

## Conclusion

This study provided descriptive information on the bout outcomes, and the background and characteristics, and punch output of boxers contesting for the World Heavyweight Championship in the past 30+ years. The USA have clearly dominated the division in this timeframe, as the most represented nation and producing the most individual champions. However, Denmark, Ukraine and the UK are the leading nations in relation to ‘success rate’ in World Heavyweight Championship contests. A form of knockout (TKO or KO) was the most common method of victory, in addition to a large number of points decisions. Differences in select characteristics, anthropometry and punch output data was observed between winners and losers. Specifically, winners tended to be taller, possess a greater reach, and consistently threw and landed more jabs and power punches than their unsuccessful counterparts. The anthropometric differences observed between heavyweight winners and losers in the present study, highlight the potential advantages of superior height and reach. Additionally, the superior punch volume and accuracy of winners of World Heavyweight Championship contests, may have practical implications for training and tactical strategies for practitioners working with professional heavyweight boxers. The findings of the present study may also be of interest to professional boxing enthusiasts.
